# Gene-Directed Enzyme Prodrug Therapy by Dendrimer-Like Mesoporous Silica Nanoparticles against Tumor Cells

**DOI:** 10.3390/nano11051298

**Published:** 2021-05-14

**Authors:** Vicente Candela-Noguera, Gema Vivo-Llorca, Borja Díaz de Greñu, María Alfonso, Elena Aznar, Mar Orzáez, María Dolores Marcos, Félix Sancenón, Ramón Martínez-Máñez

**Affiliations:** 1 Instituto Interuniversitario de Investigación de Reconocimiento Molecular y Desarrollo Tecnológico (IDM), Universitat Politècnica de València, 46022 Valencia, Spain; vicanno@etsid.upv.es (V.C.-N.); gevillo1@posgrado.upv.es (G.V.-L.); bordiade@upvnet.upv.es (B.D.d.G.); maalna8@upvnet.upv.es (M.A.); elazgi@upvnet.upv.es (E.A.); mmarcos@qim.upv.es (M.D.M.); fsanceno@upvnet.upv.es (F.S.); 2 Departamento de Química, Universitat Politècnica de València, Camino de Vera s/n, 46022 Valencia, Spain; 3 Unidad Mixta UPV-CIPF de Investigación en Mecanismos de Enfermedades y Nanomedicina, Universitat Politècnica de València y Centro de Investigación Príncipe Felipe, C/ Eduardo Primo Yúfera 3, 46012 Valencia, Spain; morzaez@cipf.es; 4 CIBER de Bioingeniería, Biomateriales y Nanomedicina (CIBER-BBN), 46022 Valencia, Spain; 5 Unidad Mixta de Investigación en Nanomedicina y Sensores, Instituto de Investigación Sanitaria La Fe (IISLAFE), Universitat Politècnica de València, Avda Fernando Abril Martorell, 46026 Valencia, Spain; 6 Centro de Investigación Príncipe Felipe, Laboratorio de Péptidos y Proteínas, C/ Eduardo Primo Yúfera 3, 46012 Valencia, Spain

**Keywords:** GDEPT, DMSNs, tumor treatment, drug delivery

## Abstract

We report herein a gene-directed enzyme prodrug therapy (GDEPT) system using gated mesoporous silica nanoparticles (MSNs) in an attempt to combine the reduction of side effects characteristic of GDEPT with improved pharmacokinetics promoted by gated MSNs. The system consists of the transfection of cancer cells with a plasmid controlled by the cytomegalovirus promoter, which promotes β-galactosidase (β-gal) expression from the bacterial gene lacZ (CMV-lacZ). Moreover, dendrimer-like mesoporous silica nanoparticles (DMSNs) are loaded with the prodrug doxorubicin modified with a galactose unit through a self-immolative group (DOXO-Gal) and modified with a disulfide-containing polyethyleneglycol gatekeeper. Once in tumor cells, the reducing environment induces disulfide bond rupture in the gatekeeper with the subsequent DOXO-Gal delivery, which is enzymatically converted by β-gal into the cytotoxic doxorubicin drug, causing cell death. The combined treatment of the pair enzyme/DMSNs-prodrug are more effective in killing cells than the free prodrug DOXO-Gal alone in cells transfected with β-gal.

## 1. Introduction

Cancer disease causes high mortality and morbidity around the world. There are more than a hundred tumor types that can take place in every part of the body, each one with their own peculiarities. In addition, there is a large variability between cancer of different patients and even between cells in a tumor [1,2].

Nowadays, many tumor types can be efficiently cured, especially if they are diagnosed in early stages [3]. However, there are still several tumor types without a known effective treatment. Despite the fact that conventional treatments used (i.e., surgery, radiotherapy, and chemotherapy) have considerably improved over the years, they still present many drawbacks. For example, drugs used in chemotherapy have a restricted therapeutic index and high drug’s doses cannot be administered to patients without important undesired side effects. Furthermore, the pharmacokinetic profile of hydrophobic drugs is not efficient, as they tend to accumulate in adipose tissue and derived organs or to be sequestered in lipid droplets [4]. Therefore, the amount of drug that effectively reaches the tumor area is much smaller than the dosage provided and sometimes it is not high enough to be effective. Besides, the appearance of resistance is another important limitation of current cancer treatments. Tumor cells can adapt themselves and resist the treatment, for example, by overexpressing efflux pumps (multidrug resistance) or by effectively using DNA repair enzymes [5]. As a result, and despite the outstanding progresses and efforts made during the last decades, cancer treatment still remains a big challenge for scientists and medical staff from all over the world [3].

In this context, gene-directed enzyme prodrug therapy (GDEPT) has emerged as a successful new strategy in cancer treatment, enhancing efficacy, and reducing the toxicity and off-target effects of conventional treatments. GDEPT is usually comprised of a three-components system: a prodrug, a gene coding system, and a carrier. In short, it is based on the induction of a transgene expression in specific cells (through a plasmid transfection or virus transduction) in order to enzymatically activate a non-effective prodrug into an active cytotoxic drug once the carrier reaches the target cell. GDEPT technology has become a promising alternative to conventional cancer treatments in the last years and the number of systems based on GDEPT reaching clinical trials has kept at a constant growth [6]. The most reported and studied enzyme-drug pair systems using GDEPT technology are herpes simplex virus thymidine kinase/ganciclovir (HSV-TK/GCV) [7], cytosine deaminase/5-fluorocytosine (CD/5-FC) [8], cytochrome P450/cyclophosphamide or ifosfamide (CYPs/CPA or IFA) [9] or oxazaphosphorine (CYPs-OXP) [10], purine nucleoside phosphorylase/6-methylpurine deoxyriboside (PNP/MEP) [11] carboxypeptidase G2/nitrogen mustard (CPG2/NM) [12], and nitroreductase/CB1954 [13]. GDEPT has two main advantages such as specificity of gene expression just in tumor cells and the known bystander effect due to the spread of the cytotoxic drug to the surrounding cells. However, a typical low transfection expression is achieved, which must be improved in the future [14].

To overcome current GDEPT limitations, several combinations of prodrugs and nanoobjects have been described, but only using the enzyme directly to activate the prodrugs instead of their encoding gene [15,16,17,18,19]. However, to the best of our knowledge, no GDEPT system using mesoporous silica nanoparticles (MSNs) as a carrier has been reported to date. MSNs have been widely studied in the last years in biomedical applications due to their exclusive characteristics such as high specific surface area, tunable morphology and pore size, and large drug loading capacity. Moreover, silica has an excellent biocompatibility, inertness, and easy surface functionalization [3,20,21]. In some reported examples, MSNs are functionalized with molecular gates, which provide on-command control release and this has boosted their use as smart nanodevices within the nanomedicine field [22,23,24,25] in many applications like drug release [26,27], gene and RNA delivery [28,29], immunotherapy [30,31], bioimaging [32,33], biomarkers sensing or diagnostic [34,35,36,37,38], tissue engineering [39,40], theragnostics [41,42], and communication protocols [43,44,45,46]. MSNs as carriers improve the pharmacokinetics of the drugs by different phenomena. On one hand, they increase the cell uptake, helping drugs to cross the cell membrane through endocytosis [21]. Additionally, the encapsulation protects the drug from unspecific degradation in the organism, reducing its undesired toxicity. In particular, prodrugs encapsulation is an important issue to increase their efficiency and to avoid their accumulation in normal organs. Although prodrugs efficiency has been found to be low in normal conditions, its occasional activation could represent a significant increase in side toxicity [14,47]. Nanocarriers also increase the bioavailability and biodistribution, especially of hydrophobic drugs, reducing the limitations derived from the low solubility in water [48,49]. Finally, MSNs can be functionalized with antibodies, peptides, aptamers, or other biomolecules in order to selectively target specific cell receptors [21,23]. As a result, the amount of prodrug reaching the target cells can increase as well as the effectiveness of the treatment and the therapeutic index.

**Scheme 1:**
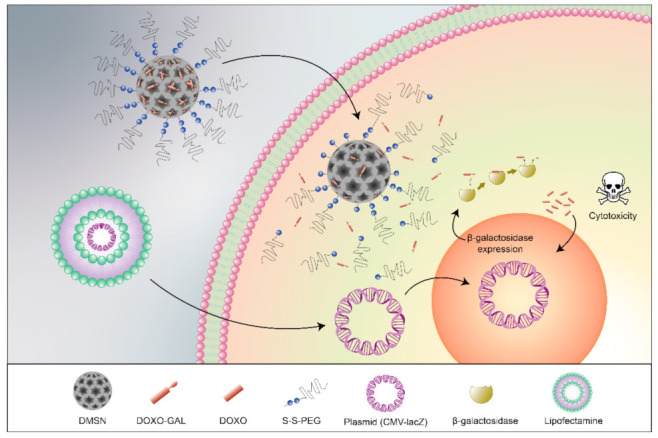
Scheme of the mechanism of action of the GDEPT system. Cancer cells are transfected with the plasmid CMV-lacZ to promote β-gal expression. Then, β-gal-expressing cells are treated with DOXO-Gal@DMSN-PEG, which specifically release the prodrug in the cytoplasm of cells. DOXO-Gal is activated after the enzymatic activity of β-gal, yielding the cytotoxic drug doxorubicin and causing cell death.

Based on the above, the aim of the present work was to demonstrate the use of MSNs as suitable carriers in GDEPT therapy. The system we developed consist of the expression in cells of the enzyme β-gal, whose expression in healthy and untreated cancer cells are negligible, and the use of dendrimer-like mesoporous silica nanoparticles (DMSNs). DMSNs are loaded with doxorubicin modified with a galactose unit through a self-immolative group (DOXO-Gal) and capped with a disulfide-containing polyethyleneglycol gatekeeper. We demonstrate that a reducing environment induces disulfide bond rupture in the gatekeeper with the subsequent DOXO-Gal delivery. Moreover, DOXO-Gal, which does not present cytotoxicity, is enzymatically converted by β-gal into the cytotoxic doxorubicin (Scheme 1). This GDEPT technology, based in the enzyme-prodrug pair β-gal/DOXO-Gal using DMSNs as carriers, was validated in vitro in LN18 glioblastoma cells.

## 2. Materials and Methods

### 2.1. Materials

Acetobromo-α-D-galactose, β-galactosidase from Aspergillus oryzae (β-gal), cetyltrimethylammonium tosylate (CTATos), 2,2′-Dipyridyl disulfide, Dulbecco’s Modified Eagle’s Medium (DMEM), L-glutathione reduced (GSH), 4-hydroxibenzaldehyde, 3-mercaptopropyltrimetoxysilane (MPTMS), 4-nitrophenylchloroformate, [Ru(bpy)_3_]Cl_2_ (Rubpy), sodium borohydride, tetraethylorthosilicate (TEOS), triethanolamine (TEAH_3_), and trimethylamine were purchased from Sigma-Aldrich (Sigma-Aldrich Química S.L., Madrid, Spain). mPEG-SH 5k (PEG-SH) was purchased from Biochempeg (Biochempeg Scientific Inc., Watertown, MA, USA). Doxorubicin hydrochloride (DOXO·HCl) was purchased from Carbosynth (Carbosynth Ltd., Compton, Berkshire, UK). Acetone, acetonitrile, dichloromethane, disodium hydrogen phosphate, isopropyl alcohol, potassium chloride, potassium dihydrogen phosphate, potassium carbonate, sodium chloride, sodium dihydrogen phosphate, and sodium hydroxide were provided by Scharlab (Scharlab S.L., Barcelona, Spain). The WST-1 assay kit was provided by Roche Applied Science (Roche Molecular Systems Inc., Madrid, Spain). Lipofectamine 3000 Reagent was purchased to Invitrogen (Thermo Fisher Scientific Inc., Madrid, Spain). Plasmid pCMV-SPORT-βgal was supplied by Life Technologies (Thermo Fisher Scientific Inc., Madrid, Spain). Opti-MEM^®^ I (1×), penicillin, and streptomycin (Pen Strep) were purchased from Gibco (Thermo Fisher Scientific Inc., Madrid, Spain).

### 2.2. General Techniques

Transmission electron microscopy (TEM) representative pictures were captured using a JEOL JEM-1010 (100 kV) microscope (JEOL Europe SAS, Croissysur-Seine, France). For sample visualization, a suspension of 1 mg mL^−1^ in distilled water was prepared and placed on carbon film supported copper electron microscopy grids. Samples were left drying for at least 24 h. To obtain the powder x-ray diffraction (PXRD) data, an AXS D8 Advance diffractometer from Bruker (Bruker, Coventry, UK) with Cu-Kα radiation was used. Dynamic light scattering (DLS) was measured using a ZetaSizer Nano ZS Instrument from Malvern (Malvern Panalytical Ltd, Malvern, UK). To evaluate the particle size in solution, a 633 nm laser was used, and the signal was collected at 173°. Particle suspensions (0.1 mg mL^−1^) were prepared using distilled water (dH_2_O). Thermogravimetry of the materials was performed using a TGA/SDTA 851e balance from Mettler Toledo (Mettler Toledo Inc., Schwarzenbach, Switzerland). Loss weight in an oxidant atmosphere (air, 80 mL·min^−1^) was registered within a dynamic step in which was applied an increase of 10 °C min^−1^ in the interval from 20 °C to 1000 °C. Then, temperature was maintained at 1000 °C for an extra 5 min. Porosimetry studies were performed using nitrogen and Tristar II Plus equipment from Micromeritics (Micromeritics Instrument Corporation, Norcross, GA, USA). Sample degasification was performed overnight at 90 or 120 °C. Specific surface area of each sample was calculated from the adsorption data by applying the BET (Brunauer–Emmett–Teller) model. The BJH (Barrett–Joyner–Halenda) model was used to determine pore size and volume. Spectroscopy fluorescence measurements were recorded using a FP-8500 spectrometer from JASCO (JASCO, Easton, OH, USA). The used wavelengths were: [Ru(bpy)_3_]Cl_2_ (*λ*_ex_. = 570 nm, *λ*_em_. = 594 nm) and DOXO/DOXO-Gal (*λ*_ex_. = 495 nm, *λ*_ex_. = 554 nm). Finally, to perform cell viability studies, a Wallac 1420 Victor2 Microplate Reader from Perkin Elmer (Perkin Elmer Inc., Walthan, MA, USA) was used.

### 2.3. Synthesis of DOXO-Gal

*N*-(α-D-galactopyranosylbenzyloxycarbonyl)-doxorubicin (DOXO-Gal) was prepared according to the literature procedures reported [50] with some variations. These modifications involved the direct use of the commercially available acetobromo-α-D-galactose **2** attending to the experimental conditions reported by Ferrari et al. [51], substitution of pyridine with triethylamine as a base, and final deacetylation of DOXO-Gal with K_2_CO_3_ rather than NaOMe. See the Supplementary Materials for synthetic conditions and chemical characterization details of the prodrug DOXO-Gal and its intermediates.

### 2.4. Synthesis of DMSNs

DMSNs were synthesized according to the Yu et al. method [52]. Briefly, 0.96 g of CTATos (2.13 mmol) were dissolved in a 100 mL round bottomed flask using 50 mL of deionized water. Then, 0.52 mmol of TEAH_3_ (74 mg) was added. The solution was maintained for 1 h at 80 °C under stirring. Afterward, 40.1 mmol of TEOS (7.8 mL) was rapidly added, and a white precipitate appeared. After 2 h, heating was stopped. When the mixture was at room temperature, the obtained solid was vacuum filtered using a Büchner funnel provided with a paper filter (Grade 3MM CHR). The solid was washed thoroughly with water until neutral pH. Finally, the solid was dried under vacuum for 12 h to obtain 2.3 g of DMSN as a white powder. The template phase was removed in an oxidizing atmosphere at 550 °C for 5 h. A total of 1.1 g of calcined DMSN was finally obtained.

### 2.5. Synthesis of DOXO-Gal@DMSN-PEG

First, 100 mg of DMSN was loaded with 105 mg of DOXO-Gal (1.25 mmol prodrug/g NP). The mixture was suspended in 15 mL of methanol using an ultrasonic bath. Then, the suspension was maintained for 48 h at room temperature under stirring. In a following step, the resulting material was vacuum filtered over a #5-pore size fritted funnel, and dried under vacuum overnight, obtaining 184 mg of DOXO-Gal@DMSN. The further functionalization stage was based on a previously reported procedure [53]. In short, a suspension of 100 mg of DOXO-Gal@DMSN in 5 mL of acetonitrile was briefly sonicated until the solid was finely dispersed. Then, 186 µL of MPTMS was added and the mixture was maintained for 5.5 h at room temperature under stirring. Finally, 2,2′-dipyridyl disulfide (220 mg) was added. Suspension was stirred overnight at room temperature and then vacuum filtered over a #5-pore size fritted funnel. A total of 5 mL acetonitrile was added to remove the excess of 2,2′-dipyridyl disulfide. After drying, the solid was ground and weighed. A total of 93 mg of material was obtained. In a further step, 50 mg of the thiol-modified material and 120 mg of PEG-SH were resuspended and briefly sonicated in 5 mL of acetonitrile until the solid was finely dispersed. After stirring overnight at room temperature, the obtained solid was filtered over a #5-pore size fritted funnel and washed first with acetonitrile, then with abundant water, and finally with PBS to remove the excess of DOXO-Gal. A total of 29 mg of DOXO-Gal@DMSN-PEG were obtained after drying under vacuum.

Negative control nanoparticles (DMSN-PEG) were synthesized in parallel to the experimental procedure described above, with the only difference that no DOXO-Gal was loaded in them. These nanoparticles were used to calculate the nanoparticle payload. Additionally, nanoparticles Rubpy@DMSN-PEG were also synthesized in parallel, in which capped nanoparticles were loaded with [Ru(bpy)_3_]Cl_2_. These were used in the optimization of the working conditions of the gatekeeper and to perform some related characterization measurements. See Supplementary Materials for the Rubpy@DMSN-PEG characterization details.

### 2.6. Release Assays

#### 2.6.1. [Ru(bpy)_3_]Cl_2_ Release

A total of 0.5 mg of Rubpy@DMSN-PEG was placed in a microtube and suspended in 2 mL of PBS. Then, the suspension was equally divided into two Eppendorf tubes. Suspensions were maintained under stirring at 37 °C for 3 h. A total of 1 mL of a 1 mM solution in water of GSH was prepared, and 10 µL was added over one of the tubes up to a final concentration of 10 µM, with the addition time of GSH as 0 considered. Different aliquots of 130 µL were taken over time for each tube, centrifuged (5 min, 13,500 rpm) to eliminate the nanoparticles, and the supernatant’s fluorescence was measured (*λ*_ex_. = 570 nm, *λ*_em_. = 594 nm).

#### 2.6.2. Forced Release

A total of 0.1 mg of DOXO-Gal@DMSN-PEG was suspended in DMSO (1 mL) and stirred for 1 h at 37 °C. Afterward, the suspension was centrifuged, and the supernatant was measured by fluorescence spectroscopy (*λ*_ex_. = 495 nm, *λ*_ex_. = 554 nm). The supernatant was properly diluted to fall into the linear region of the calibration curve of DOXO-Gal in DMSO prepared previously. Nanoparticles were suspended and stirred with DMSO as often as necessary until the nanoparticles mostly lost their color. Finally, the successive supernatants were summed.

### 2.7. Cells Lines and Maintenance

LN18 glioblastoma cells used for the study were kindly provided by the Signaling and New Therapeutic Targets research group (SINDATER, Barcelona, Spain), led by Dr. Víctor Yuste. Cells were incubated in high-glucose DMEM with 10% of serum fetal bovine and in 5% CO_2_ at 37 °C and underwent passage twice within a week.

### 2.8. Transformation, Cloning, and Extraction

*E. coli* DH5α was transformed to clone the purchased plasmid (pCMV-SPORT-βgal), which was grown in lysogeny broth (LB) and ampicillin. A QIAGEN (QIAGEN, Hilden, Germany) Plasmid Midi Kit was used to extract the plasmid and an electrophoretic assay was performed to check the good conditions of the plasmid.

### 2.9. β-Gal Activity Assay

The β-gal staining assay was carried out in LN18 cells using a kit from Cell Signaling (Cell Signaling Technology Inc., Danvers, MA, USA). Cells were assayed overnight at 37 °C in the absence of CO_2_, as per the manufacturer’s instructions. At least 1000 cells were counted to determine the transfection efficiency, which was calculated bearing in mind the number of positive cells (blue) and the number of total cells in the culture.

### 2.10. Transfection Assays

LN18 cells were transfected with lipofectamine 3000 reagent according to the manufacturer’s protocols, in a 24-well plate. Briefly, lipofectamine (0.5 µL) was dissolved with 50 µL of Opti-MEM and left to stand for 5 min. Then, a solution of 0.2 µg of plasmid in 50 µL of Opti-MEM was added. The mixture was incubated for a further 20 min. Then, the obtained solution was deposited in the corresponding well containing 150 µL of Opti-MEM. Cells were incubated for 4 h at 37 °C. In a final step, the medium was exchanged by 500 µL of DMEM.

### 2.11. Treatments with DMSN

Different (25 and 50 µg/mL) suspensions of DMSNs in DMEM were used to treat the cells. A total of 2% Pen Strep antibiotic solution was added to avoid potential contaminations. The treatment with DMSNs was performed the day after the transfection. Medium was replaced 24 h after the treatment to remove excess DMSNs.

### 2.12. Viability Assays

Viability assays were performed 72 h after treatment with DMSNs. Each well of a 24-well microtiter plate was assayed with 50 µL of WST-1 reagent incubated within 1 h at 37 °C. Then, cell activity was calculated using the absorbance at 440 nm.

## 3. Results and Discussion

### 3.1. Synthesis and Characterization

**Scheme 2:**
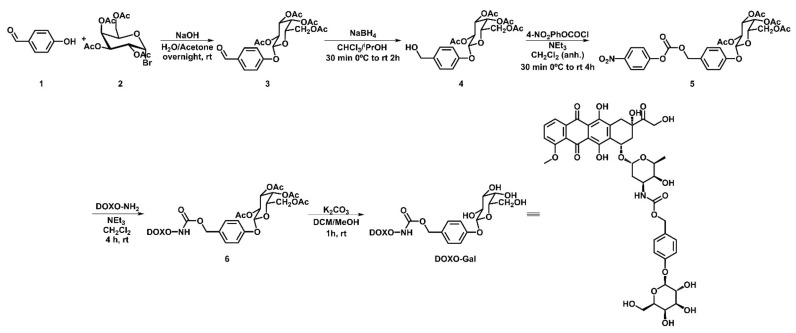
Synthesis steps to obtaining the prodrug DOXO-Gal.

**Scheme 3:**
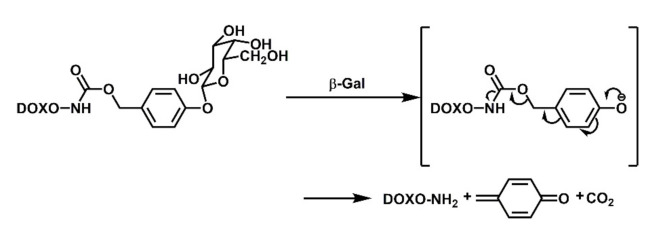
Mechanism of DOXO release after the enzymatic action of β-gal.

DOXO-Gal was obtained according to the synthetic path depicted in Scheme 2, following the procedure described by [50] with some variations (described in the Supplementary Materials). In short, compound **3** was first synthesized by the condensation under basic conditions of the commercial phenol **1** and the galactose derivative **2** (44% yield). Reduction of the aldehyde **3** with sodium borohydride afforded benzyl alcohol 4 in quantitative yield. Treatment of **4** with *p*-nitrophenyl chloroformate and triethylamine led to **5** (64% yield), which was reacted with doxorubicin to obtain **6** (62% yield). Finally, deprotection of the hydroxyl groups with potassium carbonate yielded the final prodrug DOXO-Gal in a 72% yield. This product releases the active drug doxorubicin in the presence of β-gal, which promotes the enzymatic cleavage of the *O*-glycosidic bond in DOXO-Gal (Scheme 3).

**Scheme 4:**
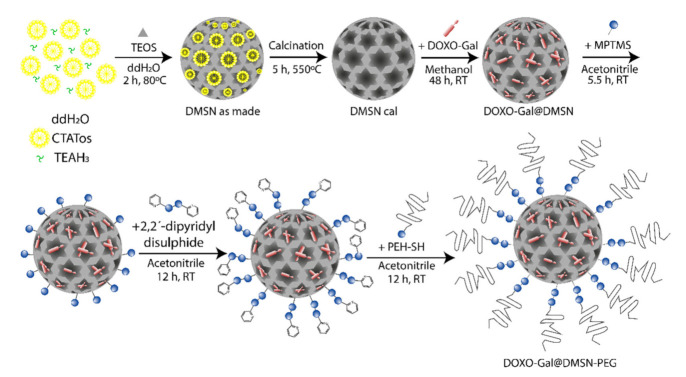
Synthetic route followed to prepare the final solid DOXO-Gal@DMSN-PEG.

Dendrimer-like MSNs used in this study (DMSNs) were prepared with the method described by Yu et al. [52]. DMSN were loaded with DOXO-Gal and the silica surface was reacted first with MPTMS, then with 2,2′-dipyridyl disulfide and finally with mPEG-SH 5k (PEG-SH) to obtain DOXO-Gal@DMSN-PEG (Scheme 4). DMSNs were used in this study, as pore dimensions must be large enough to host DOXO-Gal molecules, whose estimated size is ca. 2.7 nm. As the capping gatekeeper, a 5 kDa PEG was chosen to fully cap the pores in DMSNs. According to Wang and co-workers [54], the chain length of 5 kDa PEG is 4.5 nm, which is expected to be enough to cover the pores of DMSNs. This PEG derivative is additionally used as a gate-like platform able to allow the release of the cargo in the cells due to the high concentration of glutathione, which would result in cleavage of the disulfide bond [53]. In addition, a similar gated solid loaded with [Ru(bpy)_3_]Cl_2_ (estimated diameter ca. 1.2 nm) (Rubpy@DMSN) and unloaded PEG-capped nanoparticles (DMSN-PEG) were also prepared to perform some assays.

**Figure 1:**
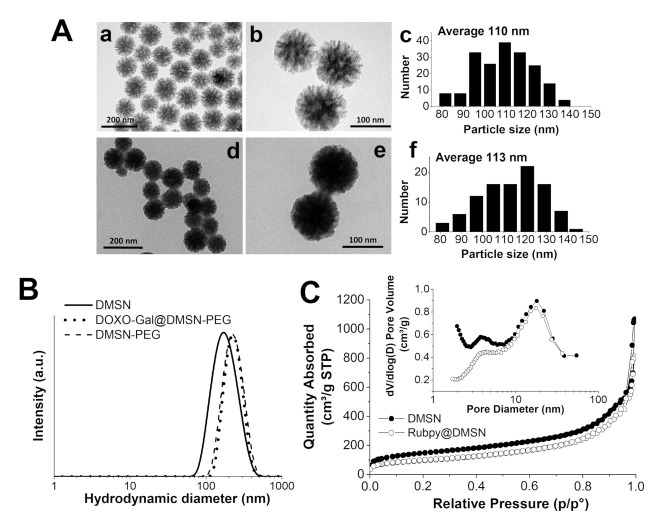
(**A**) Representative TEM images of DMSN (**a**,**b**) and DOXO-Gal@DMSN-PEG (**d**,**e**). Histogram with particle size distribution measured by TEM of DMSN (**c**) and DOXO-Gal@DMSN-PEG (**f**). Size distributions were established upon analysis of 100 nanoparticles. (**B**) Intensity PSD DLS curves of DMSN, DOXO-Gal@DMSN-PEG, and DMSN-PEG. Samples measured were suspended in distilled water. Brief spin pulses were applied in order to remove large aggregates or sediments. (**C**) N_2_ adsorption–desorption isotherms of DMSN and Rubpy@DMSN; Inset: pore-size distribution of DMSN and Rubpy@DMSN.

The nanoparticles were characterized using standard techniques. As made DMSN were analyzed by PXRD, but no peaks were observed at low angles, thus indicating that these mesoporous particles did not present an ordered pore distribution. Nevertheless, the TEM images clearly showed a dendrimer-like porous structure in spherical nanoparticles of ca. 110 nm in diameter (Figure 1A, a–c). DOXO-Gal@DMSN-PEG images (Figure 1A, d–f) displayed the same dendrimer-like structure and morphology (diameter size ca. 113 nm). In addition, the hydrodynamic diameters of calcined DMSN, DOXO-Gal@DMSN-PEG, and DMSN-PEG were determined by DLS (Figure 1B). DMSN samples showed a population centered at 170 nm. Meanwhile, the hydrodynamic diameter of DOXO-Gal@DMSN-PEG and DMSN-PEG were 225 nm and 223 nm, respectively. As expected, the hydrodynamic diameter measured was slightly higher than the size observed using TEM, which was caused by the hydrodynamic sphere that diffuses around the nanoparticles. Accordingly, functionalization with PEG polymer promotes an increase in the solvation layer around the polymer, making the hydrodynamic diameter increase.

Furthermore, N_2_ adsorption-desorption isotherms of calcined DMSN and Rubpy@DMSN were recorded for characterizing the textural properties of these solids (Figure 1C). DOXO-Gal@DMSN was not studied due to the toxicity of the cargo, and DMSN nanoparticles capped with PEG and loaded with [Ru(bpy)_3_]Cl_2_ as a model cargo (Rubpy@DMSN) were used instead. The obtained data for DMSN corresponded to a type IV isotherm, indicating the existence of mesopores. The Rubpy@DMSN isotherm presented a decrease in the nitrogen absorption capacity in comparison to the DMSN isotherm due to the presence of the [Ru(bpy)_3_]Cl_2_ cargo. In this context, a specific surface area of 523.3 m²/g was calculated for DMSN and 343.7 m²/g for Rubpy@DMSN. The pore volume of DMSN and Rubpy@DMSN were respectively 0.93 cm³/g and 0.71 cm³/g, according to the BJH method. A bimodal distribution of pore sizes was observed centered at 4.0 nm and 17.8 nm (Figure 1C, inset). It is noticeable how cargo loading mostly affected pores at 4.0 nm and not the larger ones. A similar behavior has also been observed in previous works [55]. Moreover, a sharp step at P/P_0_ > 0.9 could also be observed in both isotherms, which corresponded to interparticle porosity.

In order to characterize the functionalization and loading capacity, DMSN, DOXO-Gal@DMSN-PEG, and DMSN-PEG were analyzed by thermogravimetry (Figure S2). DMSN showed a 2.3% of dry weight loss, which can be attributed to silanol condensation. Taking this into consideration, from these studies, a total content of organic matter of 24.5% and 13.6% was found for DOXO-Gal@DMSN-PEG and DMSN-PEG, respectively. Considering these values, it is possible to estimate the amount of prodrug DOXO-Gal loaded in DOXO-Gal@DMSN-PEG as 10.9% (109 µg/mg of nanoparticle). The amount of loaded cargo was also determined with a forced extraction of DOXO-Gal from DOXO-Gal@DMSN-PEG with DMSO (see the Materials and Methods section for details). According to this experiment, the total amount of DOXO-Gal extracted was 78 µg/mg of nanoparticles (7.8%). This result was slightly lower than that obtained with the TG analysis (a 3.1% difference) and tentatively attributed to some DOXO-Gal strongly adsorbed onto the surface of the silica nanoparticles that could not be extracted with DMSO. 

**Figure 2:**
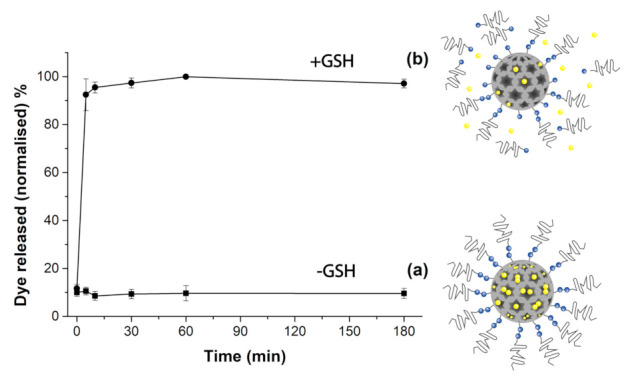
Release profile of [Ru(bpy)_3_]^2+^ from Rubpy@DMSN-PEG in the absence (**a**) and presence (**b**) of GSH 10 µM.

The DOXO-Gal prodrug has been previously studied in systems where β-gal is naturally overexpressed [56,57]. Moreover, we also demonstrated the use of other galactose-functionalized prodrugs based on Navitoclax for selectively killing senescent cells, which overexpress β-gal [58], galactose-functionalized dyes for senescent cell detection [59,60,61,62] or the use of galactose-capped nanoparticles for programmed cargo delivery in senescent cells taking advantage of the overexpression of β-gal [63,64]. In addition, the pair β-gal/DOXO-Gal has previously been successfully reported in the literature [65]. Based on these previous results, on-command cargo delivery from the nanoparticles in the presence of β-gal was studied. For this study, we used Rubpy@DMSN-PEG rather than DOXO-Gal@DMSN-PEG due to the low solubility of DOXO-Gal in water that inhibited us from obtaining reliable release curves. The experiment aimed to demonstrate the ability of the PEG gatekeeper to maintain undelivered a certain cargo. GSH was used to simulate the reducing environment present in cells, which should induce rupture of the disulfide bond, resulting in cargo delivery. Figure 2 confirms that a rapid and effective delivery was registered immediately after the addition of GSH in accordance with results previously reported using disulfide-PEG gatekeepers [53,66]. In contrast, in the absence of GSH, release was very low, demonstrating the ability of the PEG polymer to block the pores.

### 3.2. Cells Studies

**Figure 3:**
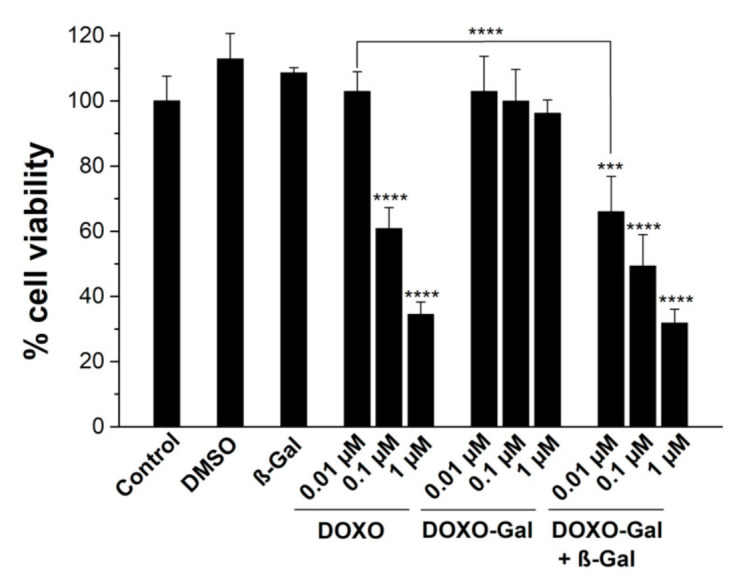
Cytotoxicity profile of the LN18 cell line treated with free DOXO, DOXO-Gal, and DOXO-Gal + β-gal at different concentrations. DOXO and DOXO-Gal were dissolved in DSMO and diluted with DMEM to a final DMSO volume percentage of 0.3%. β-gal was used at 1 mg/mL. Cell viability was studied using WST-1 at 72 h. In this study, the statistical significance was determined by one-way ANOVA and Dunnet post-test (*** *p* < 0.0002, **** *p* < 0.0001).

First, we validated the non-toxicity of DOXO-Gal and the ability of β-gal to activate the prodrug. DOXO-Gal, DOXO, β-gal, and β-gal + DOXO-Gal were added to LN18 cell cultures and the results are gathered in Figure 3. The exposure of cells to DMEM with DMSO 0.3%, which was employed to dissolve DOXO or DOXO-Gal, was not toxic when compared with the untreated cells (control). A total of 1 mg/mL β-gal was also tested in cells and no toxicity was found. Once toxicity of the medium and enzyme was discarded, different concentrations of DOXO were evaluated (0.01, 0.1, and 1 µM), obtaining an increased toxicity when the DOXO amount increased. A low toxicity was found when cells were treated with DOXO 0.01 µM. However, only 60% and 35% of cells survived in the presence of DOXO 0.1 µM and 1 µM, respectively. No toxicity was found when cells were treated separately with DOXO-Gal at 0.01, 0.1, and 1 µM. However, when the cells were exposed to both DOXO-Gal and β-gal, cell viability decreased, showing a similar tendency to that for DOXO; 66%, 50%, and 32% of cells survived in the presence of DOXO-Gal 0.01, 0.1, and 1 µM, respectively, when β-gal was also added at 1 mg/mL. At lower concentrations, the combination of DOXO-Gal + β-gal showed a higher toxicity compared to DOXO. This might be due to differences in the compound solubility, since no cytotoxicity was reported regarding cleavage subproducts [67,68]. In summary, the use of DOXO-Gal as a prodrug was validated in LN18 glioblastoma cells. DOXO-Gal was activated by β-gal, showing a similar behavior than free DOXO upon β-gal hydrolysis.

**Figure 4:**
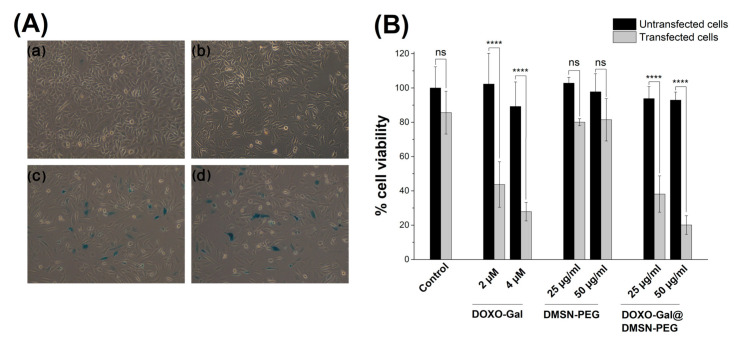
(**A**) Optical microscope images of X-Gal assay in untransfected (**a**,**b**) and transfected (**c**,**d**) LN18 cells. (**B**) Cytotoxicity profile when transfected (grey columns) and untransfected (black columns) cells were treated with free DOXO-Gal, DMSN-PEG, or DOXO-Gal@DMSN-PEG at different concentrations. DOXO-Gal was dissolved in DMSO and diluted with DMEM to a final DMSO volume percentage of 0.3%. Cell viability was studied by WST-1 at 72 h. Statistical significance was determined by one-way ANOVA and Dunnet post-test (**** *p* < 0.0001; ns = no significance).

In addition to previous studies, further studies in LN18 cells were carried out with the DOXO-Gal@DMSN-PEG nanoparticles. Effective cellular uptake of DMSN [69,70] and MSN functionalized with PEG [53] is well established in the literature. LN18 cells were transfected with the plasmid pCMV-SPORT-βgal using lipofectamine (see Materials and Methods section for details). The transfection efficiency was determined by performing an X-Gal staining assay, which determined β-gal activity after transfection. It must be noted that non-senescent LN18 cells used in these experiments do not express β-gal. The optical microscopy images on Figure 4A show the staining result before and after cells were transfected. Control cells were not stained, whereas upon transfection, certain LN18 cells were found to be blue stained, thus indicating they express β-gal. From these images, a transfection efficiency of 10% was calculated.

Taking into account the result above, we studied the ability of cells transfected by β-gal to activate the prodrug, both encapsulated (DOXO-Gal@DMSN-PEG) and free (DOXO-Gal). Transfected and untransfected cells were treated with free DOXO-Gal, DMSN-PEG, and DOXO-Gal@DMSN-PEG. Free DOXO-Gal was used as a control to determine the efficiency of the encapsulated system (i.e., DOXO-Gal@DMSN-PEG), while DMSN-PEG at the same concentrations as DOXO-Gal@DMSN-PEG were used to demonstrate the biocompatibility of the nanocarrier. Considering the data obtained by forced extraction of the prodrug from DOXO-Gal@DMSN-PEG, equivalent amounts of free DOXO-Gal were used (2 and 4 µM, respectively). Figure 4B shows the results obtained. Untransfected cells barely manifested sensitivity to the treatments applied and their viability was no lower than 90% under any conditions. In contrast, transfected but no treated cells showed a slight decrease in viability (ca. 15%), which is attributed to the basal toxicity derived from the transfection with lipofectamine. Once transfected, cells treated with DMSN-PEG were not affected compared to the control. In contrast, when transfected cells were treated with free DOXO-Gal or DOXO-Gal@DMSN-PEG, the viability was significantly reduced. In the case of free DOXO-Gal, cell viability was 44% and 28% for the two concentrations studied in comparison with the control cells, whereas a slightly higher toxicity was found upon treatment with DOXO-Gal@DMSN-PEG, the viability found being in this case of 38% and 20%.

The results obtained indicate that the GDEPT system developed worked properly. The β-gal transfected in cells, whose presence and activity was demonstrated in Figure 4A, was able to effectively activate the DOXO-Gal prodrug in the cells. On the other hand, it is remarkable that transfected cells, even though the transfection efficiency was only 10%, showed values of viability as low as 20% in the presence of DOXO-Gal@DMSN-PEG. This phenomenon can be explained by the bystander cytotoxic effect [6,71], by which once DOXO-Gal is converted to DOXO and provokes cell death, the remaining DOXO generated can be expulsed to the surrounding media and affect neighboring cells, causing a further cytotoxic effect, which has already been reported in LN18 cells [72,73]. In addition, the presence of DOXO at low concentrations in untransfected cells, due to the bystander effect, could trigger senescence [74] (senescent cells overexpress β-gal), and induce a synergistic effect together with β-gal transfection. Then, even though the transfection efficiency can be low, the GDEPT system is not strongly limited because of this reason. Finally, it could be observed how encapsulated DOXO-Gal was slightly more effective than free DOXO-Gal (in the range of 6% to 8%). This fact can be attributed to several reasons including a selective delivery of the encapsulated cargo, which was triggered by the high concentration of GSH in cells and the use of MSNs as a carrier of the poorly water soluble DOXO-Gal prodrug. In addition, encapsulating DOXO-Gal in MSN opens the way to functionalizing their surface with antibodies or other targeting ligands, which is a known approach to improve the pharmacokinetics and selectivity of drugs. Being demonstrated here the potential use of mesoporous silica nanoparticles in GDEPT systems, further in vivo studies will be carried out in the future. Moreover, the application of control release systems such as gated MSN in GDEPT opens the way to improve the latter in different ways. On one hand, and as stated above, the use of carriers such as MSN allows the easy use of targeting agents such as antibodies, peptides, or aptamers [75,76] to selectively drive the load to the desired sites. On the other hand, nanocarriers usually admit multi-functionalization [77,78], and therefore the possible inclusion of the plasmid and prodrug in the same nanodevices, simplifying the system and improving the selectivity of the GDEPT approach. Last but not least, the possible use of plasmids with a tumor specific promoter (TSP) would increase the selectivity over healthy cells and tissues, reducing the side effects of chemotherapy.

## 4. Conclusions

In summary, we developed a combined system following a GDEPT approach (β-gal and DOXO-Gal as an enzyme-prodrug pair) and the encapsulation of the prodrug (DOXO-Gal) in dendrimer-like MSN, which were additionally capped with a disulfide-containing polyethyleneglycol gatekeeper. The prodrug DOXO-Gal remained in the DMSNs until the addition of GSH, which promoted the rupture of the disulfide bonds and subsequent cargo release. DOXO-Gal prodrug activation by β-gal was validated in both β-gal added externally and in transfected β-gal cells. In cells, once the DOXO-Gal is delivered, it is converted by β-gal into the cytotoxic doxorubicin drug, causing cell death. Moreover, the DOXO-Gal-loaded and capped nanoparticles (DOXO-Gal@DMSN-PEG) demonstrated a selective toxicity to β-gal transfected cells. The combined treatment of the pair enzyme/DMSNs-prodrug resulted in a more effective effect than that observed for the free prodrug DOXO-Gal alone in cells transfected with β-gal. This study opens new perspectives to improve the efficacy of GDEPT therapy by increasing the quantity of drug that reaches the tumor cells via the use of gated DMSNs loaded with selected prodrugs.

## Data Availability

The data presented in this study are available on request from the corresponding author.

## Supplementary Materials

The following are available online at https://www.mdpi.com/article/10.3390/nano11051298/s1, Synthesis of DOXO-Gal, Figure S1: ^1^H-NMR spectrum of DOXO-Gal DMSO-d_6_; Table S1: ^1^H-NMR data of DOXO-Gal DMSO-d_6_; Figure S2: Thermogravimetric analysis of DMSN-PEG and DOXO-Gal@DMSN-PEG; Figure S3: TEM images, intensity PSD DLS curve, and thermogravimetric analysis of Rubpy@DMSN-PEG.
