# Supplementary material for: Gene-Directed Enzyme Prodrug Therapy by Dendrimer-Like Mesoporous Silica Nanoparticles against Tumor Cells
Source: Nanomaterials (Basel). 2021 May 14;11(5):1298. doi: 10.3390/nano11051298 (PMC8156333; doi:10.3390/nano11051298)
Supplement: Supplementary file 1 [file nanomaterials-11-01298-s001.zip › nanomaterials-1203524-supplementary.pdf]

Gene-Directed Enzyme Prodrug Therapy by Dendrimer-Like Mesoporous Silica Nanoparticles against Tumor Cells

Supplementary Material

Vicente Candela-Noguera [1,2,3], Gema Vivo Llorca [1,2,3], Borja Díaz de Greñu [1,2], María Alfonso [1,2], Elena Aznar [1,3,4,5], Mar Orzáez [3,6], María Dolores Marcos [1,2,3,4,5], Félix Sancenón [1,2,3,4,5] and Ramón Martínez-Máñez [1,2,3,4,5,*]

[1] Instituto Interuniversitario de Investigación de Reconocimiento Molecular y Desarrollo Tecnológico (IDM), Universitat Politècnica de València, 46022 Valencia, Spain; vicanno@etsid.upv.es (V.C.-N.); gevillo1@posgrado.upv.es (G.V.L.); bordiade@upvnet.upv.es (B.D.d.G.); maalna8@upvnet.upv.es (M.A.); elazgi@upvnet.upv.es (E.A.); mmarcos@qim.upv.es (M.D.M.); fsanceno@upvnet.upv.es (F.S.)
[2] Departamento de Química, Universitat Politècnica de València, Camino de Vera s/n, 46022 Valencia, Spain
[3] Unidad Mixta UPV-CIPF de Investigación en Mecanismos de Enfermedades y Nanomedicina, Universitat Politècnica de València y Centro de Investigación Príncipe Felipe, C/ Eduardo Primo Yúfera 3, 46012 Valencia, Spain; morzaez@cipf.es (M.O.)
[4] CIBER de Bioingeniería, Biomateriales y Nanomedicina (CIBER-BBN),46022,Valencia, Spain
[5] Unidad Mixta de Investigación en Nanomedicina y Sensores, Instituto de Investigación Sanitaria La Fe (IISLAFE), Universitat Politècnica de València, Avda Fernando Abril Martorell, 46026 Valencia, Spain
[6] Centro de Investigación Príncipe Felipe, Laboratorio de Péptidos y Proteínas, C/ Eduardo Primo Yúfera 3, 46012 Valencia, Spain
* Correspondence: rmaez@qim.upv.es (R.M.-M.)

Synthesis of DOXO-Gal

Synthesis of 4-formylphenyl-(2, 3, 4, 6-tetra-O-acetyl-B-D-galactopyranoside) **3**:

4-Hydroxybenzaldehyde **1** (244 mg, 2.0 mmol) was dissolved in 7 mL of a 0.286 M solution of aqueous NaOH (80 mg, 2.0 mmol) and cooled down in an ice bath. Acetobromo-α-D-galactose **2** (1033 mg, 2.5 mmol) was dissolved in acetone (10 mL) and added dropwise under stirring. The mixture was allowed to stand for 10 min in the ice bath and then stirred overnight at room temperature. The mixture was then placed into water (50 mL) and extracted with ethyl acetate (3 × 30 mL). The organic phase was dried with $MgSO_4$, filtered and the solvent was evaporated to dryness under reduced pressure. The crude product was purified by flash chromatography (silica, 2 × 30 cm) from hexane:ethyl acetate (7:3) to hexane:ethyl acetate (1:1), to get 4-formylphenyl-(2, 3, 4, 6-tetra-O-acetyl-B-D-galactopyranoside) **3** (400 mg, 44%) as a white solid. ^{1}H-NMR, ^{13}C-NMR and MS spectrometry were in accordance with the literature [1].

Compounds **4**, **5** and **6** were prepared according to literature procedures [1]. In the case of compound **5**, 3 equivalents of triethylamine were used as base instead of pyridine.

Synthesis of N-(α,D-galactopyranosylbenzyloxycarbonyl)-doxorubicin (**DOXO-Gal**):

Compound **6** (680 mg, 0.66 mmol) was placed in a 100 mL round bottom flask and solved in a mixture MeOH:dichloromethane (32:6 mL). A suspension of K_2CO_3 (92 mg, 0.66 mmol) in methanol was added dropwise (10 mL) and the colour changed from deep red to deep purple. The suspension was stirred for 1 h at rt. The reaction mixture was neutralized by addition of HCl in 2M in Et_2O (1 mL) and the colour turned to orange. The mixture was evaporated under reduced pressure and purified by flash chromatography (silica, 2 × 20 cm) in dichloromethane:methanol (4:1), to get N-(α,D-galactopyranosylbenzyloxycarbonyl)-doxorubicin (**DOXO-Gal**) (410 mg, 72%) as a red solid. ^{1}H-NMR, ^{13}C-NMR and MS spectrometry in accordance with literature [1].

NMR characterization of DOXO-Gal

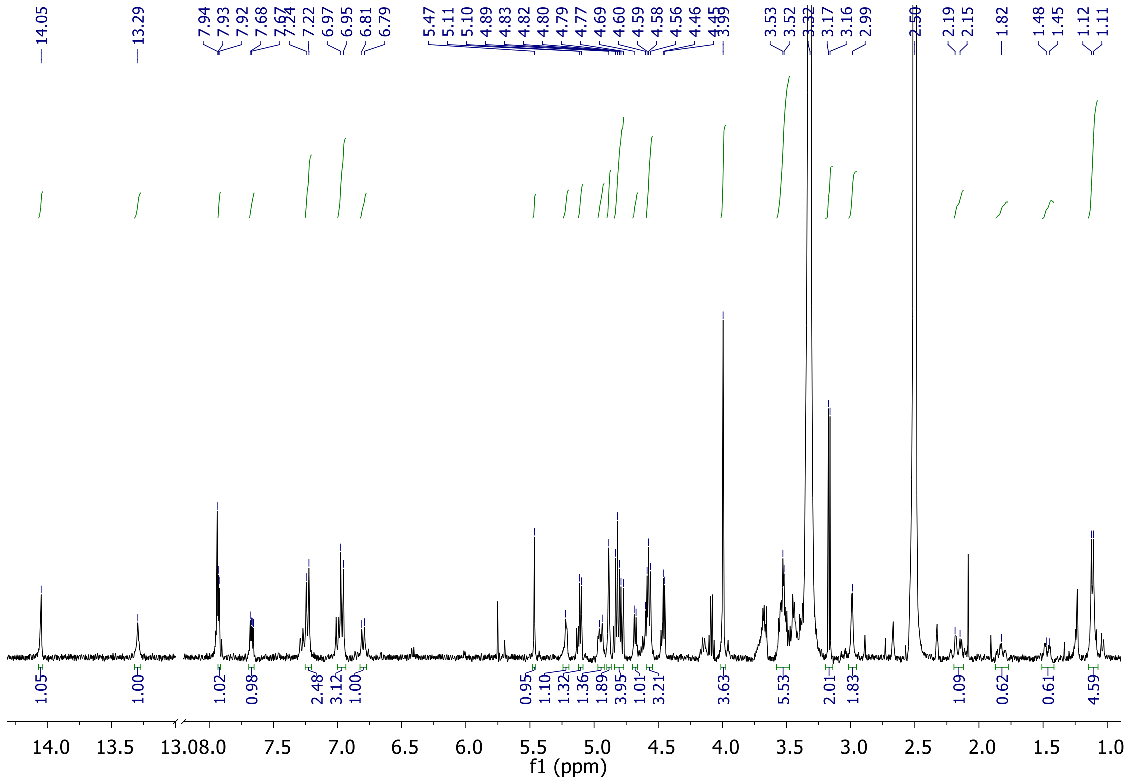

Figure S1. *^{1}H-NMR spectrum of **DOXO-Gal** in DMSO-d_6.*

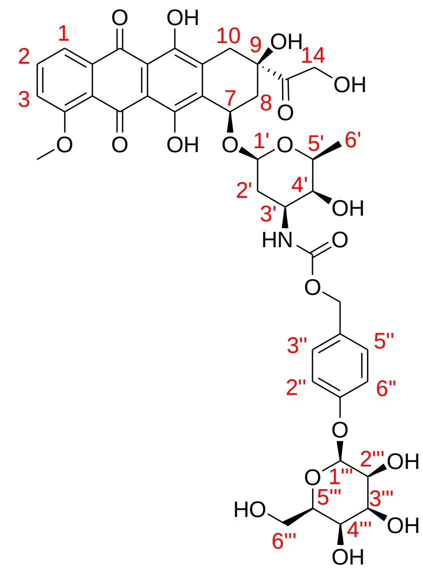

Table S1. ^{1}H-NMR data spectrum of **DOXO-Gal** in DMSO-d$_6$

(ppm)	multiplicity	Number of protons	Asignation
1.11	d	3	6′
1.43	d	1	2′
1.82	m	1	2
2.15	d	1	8
2.21	d	1	8
2.99	d	2	10
3.17-3.50	m	5	4′,5′,2′′′,3′′′,4′′′
3.53	m	1	3′
3.99	s	3	Ar-O-C<u>H</u>$_3$
4.10	m	1	5′′′
4.56-4.60	m	2	14
4.70-4.80	m	2	Ar-C<u>H</u>$_2$-O
4.82	d	1	6′′′
4.90	m	1	7
5.25	d	1	1′′′
5.45	d	1	1′
6.79	d	1	1
6.95	d	2	3′′,5′′
7.22	d	2	2′′,6′′
7.68	d	1	3
7.93	m	1	2

Nanoparticle's characterisation

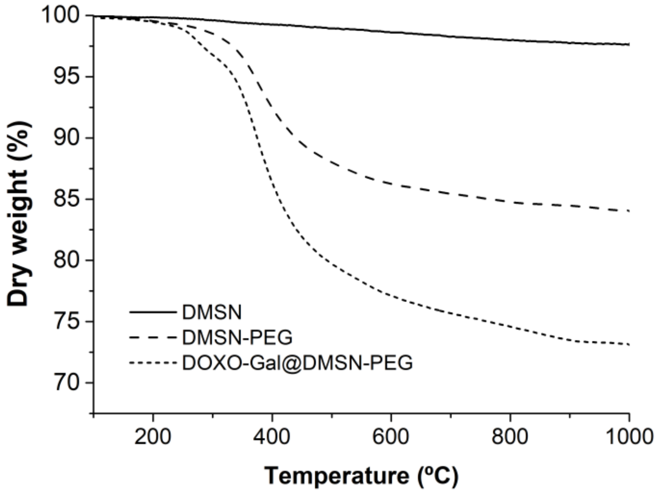

Figure S2. *Thermogravimetric analysis of DMSN,* ***DMSN-PEG*** *and* ***DOXO-Gal@DMSN-PEG***. *The percentage showed are normalised to the dry weight of each sample, which is taken at 100 °C.*

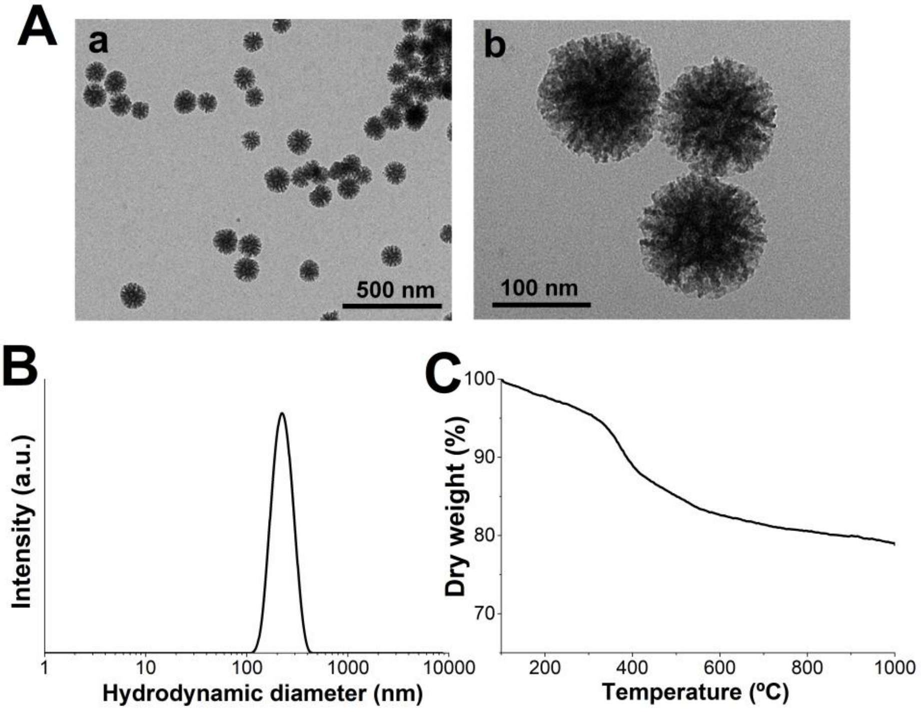

Figure S3. A) *TEM images of* ***Rubpy@DMSN-PEG*** *(**a** and **b**).* ***B)*** *Intensity PSD DLS curve of* ***Rubpy@DMSN-PEG***. *Sample measured was suspended in distilled water. Brief spin pulse was applied in order to remove large aggregates or sediments* ***C)*** *Thermogravimetric analysis of* ***Rubpy@DMSN-PEG***. *The percentage showed are normalised to the dry weight of each sample, which is taken at 100 °C.*

According to **DMSN-PEG** TG curve, the percentage of organic matter coming from $[Ru(bpy)_3]Cl_2$ is 5.2% (52 μg / mg of nanoparticle).

Reference

1. Devalapally, H.K.; Navath, R.S.; Yenamandra, V.; Akkinepally, R.R.R.; Devarakonda, R.K. β-Galactoside prodrugs of doxorubicin for application in antibody directed enzyme prodrug therapy/prodrug monotherapy, *Arch. Pharm. Res.* **2007**, *30*, 723–732, doi: 10.1007/BF02977634.
